# Use of ClearGuard HD caps in pediatric hemodialysis patients

**DOI:** 10.1007/s00467-023-06273-6

**Published:** 2024-01-25

**Authors:** Amy Nau, Troy Richardson, Diana Cardwell, Jennifer Ehrlich, Jyothsna Gattineni, Melisha Hanna, Mahima Keswani, Emily Neibauer, Kelly Nitz, Raymond Quigley, Michelle Rheault, Rebekah Sims, Mayna Woo, Bradley A. Warady

**Affiliations:** 1 Division of Pediatric Nephrology, Children’s Mercy Kansas City, Kansas City, MO USA; 2 Children’s Hospital Association, Lenexa, KS USA; 3 Division of Pediatric Nephrology, Children’s Health Dallas, Dallas, TX USA; 4 Division of Pediatric Nephrology, Stead Family Children’s Hospital, University of Iowa, Iowa City, IA USA; 5 Division of Pediatric Nephrology, Children’s Hospital Colorado, Aurora, CO USA; 6 Division of Pediatric Nephrology, Ann and Robert H. Lurie Children’s Hospital, Chicago, IL USA; 7 Division of Pediatric Nephrology, Children’s Hospital of Wisconsin, Milwaukee, WI USA; 8 Division of Pediatric Nephrology, Children’s Minnesota, Minneapolis, MN USA; 9 Division of Pediatric Nephrology, Children’s of Alabama, Birmingham, AL USA; 10 Division of Pediatric Nephrology, Stanford Medicine-Children’s Health, Lucile Packard Children’s Hospital, Palo Alto, CA USA

**Keywords:** ClearGuard, CA-BSI, Pediatric, Hemodialysis, Cap

## Abstract

**Background:**

Bloodstream infections (BSIs) are a leading cause of hospitalizations and mortality among patients receiving hemodialysis (HD) therapy, especially those with a central venous catheter (CVC) for dialysis access. The use of chlorhexidine impregnated catheter caps (ClearGuard) has been associated with a decrease in the rate of HD catheter-related BSIs (CA-BSIs) in adults; similar data have not been published for children.

**Methods:**

We compared CA-BSI data from participating centers within the Standardizing Care to Improve Outcomes in Pediatric Endstage Kidney Disease (SCOPE) collaborative based on the center’s use of ClearGuard caps for patients with HD catheter access. Centers were characterized as ClearGuard (CG) or non-ClearGuard (NCG) centers, with CA-BSI data pre- and post-CG implementation reviewed. All positive blood cultures in participating centers were reported to the SCOPE collaborative and adjudicated by an infectious disease physician.

**Results:**

Data were available from 1786 SCOPE enrollment forms completed January 2016–January 2022. January 2020 served as the implementation date for analyzing CG versus NCG center data, with this being the time when the last CG center underwent implementation. Post January 2020, there was a greater decrease in the rate of HD CA-BSI in CG centers versus NCG centers, with a decrease from 1.18 to 0.23 and 0.41 episodes per 100 patient months for the CG and NCG centers, respectively (*p* = 0.002).

**Conclusions:**

Routine use of ClearGuard caps in pediatric dialysis centers was associated with a reduction of HD CA-BSI rates in pediatric HD patients.

**Graphical abstract:**

**Figure:**
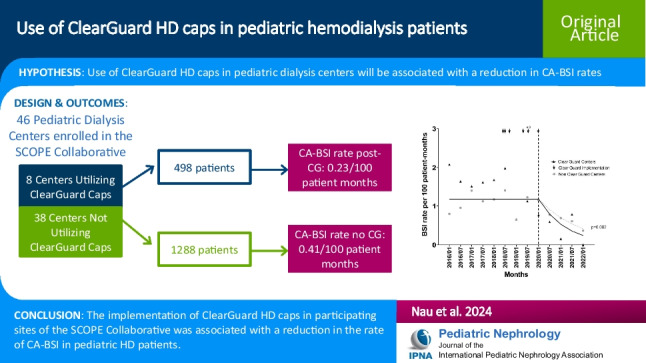
A higher resolution version of the Graphical abstract is available as Supplementary information

**Supplementary Information:**

The online version contains supplementary material available at 10.1007/s00467-023-06273-6.

## Introduction

Patients who use a central venous catheter (CVC) for hemodialysis (HD) access are at risk of experiencing a variety of complications, the most frequent of which is a catheter-associated bloodstream infection (CA-BSI). The consequences of this infection can be substantial both clinically and financially. According to the Centers for Disease Control (CDC), HD CA-BSIs are one of the leading causes of hospitalizations and mortality for HD patients [1]. In 2022, the SCOPE collaborative analyzed the fiscal implications of a CA-BSI in children receiving HD and found that, on average, each CA-BSI resulting in hospitalization incurred a cost of $18,375. Hospitalizations that required a longer stay or ICU care were associated with a significantly higher average cost [2].

Whereas the initial focus of the SCOPE collaborative was on the standardization of peritoneal dialysis (PD) catheter care practices to decrease the rate of infection in long-term pediatric PD patients, additional infection prevention strategies and quality improvement principles designed to reduce infections in pediatric HD patients were instituted in June 2013. Over the course of the collaborative, it is estimated that 862 HD access-related infections and 607 hospitalizations have been prevented and nearly $19,000,000 has been saved in participating pediatric HD patients through implementation of HD catheter care bundles [3].

There are a variety of products and practices pediatric HD programs can use for CA-BSI prevention. The ClearGuard HD cap has been designed specifically for HD catheters with a rod that extends into the catheter hub when placed on the end of a HD catheter. This rod, along with the threads on the inside of the cap, is coated with dry chlorhexidine. When the dry chlorhexidine encounters the catheter locking solution, the chlorhexidine dissolves into the locking solution. The catheter line clamps subsequently create a vestibule between the clamps and the end of the catheter filled with the antimicrobial chlorhexidine solution.

Two published multicenter adult studies have provided evidence of the benefit of ClearGuard HD caps for CA-BSI prevention in adult HD patients with a CVC as their dialysis access [4, 5]. To date, there have not been any studies regarding the use of ClearGuard HD caps in pediatric practice. We, in turn, leveraged data collected by SCOPE to evaluate the impact of this product on the rate of CA-BSIs in pediatric HD patients.

## Methods

### Study design

We performed a retrospective analysis of data submitted by centers participating in the HD arm of the SCOPE collaborative. The SCOPE collaborative is a multicenter quality improvement collaborative hosted by the Children’s Hospital Association. The collaborative design was previously described in detail by Neu et al. outlining the framework for the collaborative’s structure, purpose, and goals [6].

### Cohort and data collection

Data for this analysis were collected from patients enrolled in a SCOPE participating center from 01/01/2016 to 02/28/2022 via SCOPE enrollment forms. Enrollment forms are completed for each patient receiving care at a participating center and are submitted via an online portal. SCOPE enrollment forms are completed each time a new dialysis patient and family agree to be included in the SCOPE collaborative data collection by signing the consent/assent forms. Enrollment forms include demographic information such as dialysis modality, access type, patient age, race, gender, and cause of kidney failure. Center-specific infection data were also analyzed for the specified time frame utilizing infection data forms submitted by each center.

Information collected from each SCOPE center included the number of enrollments, median age of all enrollments, number of participants in each age range, number of participants in each gender category, number of participants in each race category, number of participants in each kidney failure cause category, and number of participants with one or more infections reported during the selected time frame. The Institutional Review Board at each center reviewed and approved the SCOPE protocol.

The SCOPE collaborative promotes standardized HD vascular access care through the implementation of specific catheter care “bundles.” These bundles have been described previously and address strategies to prevent CA-BSI in hemodialysis patients with HD catheters. HD bundle components include HD catheter dressing/site assessment, HD catheter connection, HD catheter disconnection, cap care, and HD catheter dressing change/exit care [3]. Bundle compliance is recorded and submitted for each patient and center monthly as an all or nothing characterization, meaning all bundle elements must be performed for patient care to be in compliance with the SCOPE bundle. Compliance with bundle components was reviewed for both the ClearGuard (CG) and non-ClearGuard (NCG) centers, both pre- and post-CG implementation.

### Exposure

Participating centers included in this analysis were classified as either CG or NCG centers. Those categorized as CG centers confirmed at least 2 years of ClearGuard use in > 75% of HD patients with a CVC as their dialysis access and could provide a definitive start date for ClearGuard implementation. Based on these criteria, 8 SCOPE centers were classified as CG centers and 38 were classified as NCG centers.

The timing of implementation of CG across the 8 CG centers varied from June 2018 to January 2020. We considered January 2020 as the implementation date for analyzing CG versus NCG center data, with this month being the date when the last CG center underwent implementation.

### Outcome

The primary outcome of interest was the rate of HD catheter-associated bloodstream infections (CA-BSIs). A CA-BSI was defined as a positive blood culture in a patient with a HD catheter access in the absence of another source of infection. All positive blood cultures were reported to the SCOPE collaborative and adjudicated by an infectious disease physician in accordance with the CDC guidance (“Dialysis Event Surveillance Protocol”). CA-BSI rates were calculated per 100 patient months of HD exposure.

### Statistical analysis

Categorical demographics and clinical characteristics were summarized using frequencies and percentages; continuous variables were summarized using medians and interquartile range (IQR). We compared characteristics at NCG centers versus CG centers using chi-square test for association for categorical variables and Kruskal-Wallis test for categorical variables. We assessed change in SCOPE HD bundle compliance over time with logistic regression modeling and compared changes over time between CG and NCG centers with a CG-by-time interaction effect in our model. We modeled CA-BSI rates over time using Poisson generalized linear mixed modeling (GLMM) approach assuming a natural log link function. All GLMMs included a random center effect to account for clustering of HD patients within the same center. Using these GLMMs, we performed a difference-in-difference analysis to compare serial trends in BSI rate over time between CG and NCG centers, using January 2020 as the onset of CG implementation. All analyses were performed using SAS v9.4 (Cary, NC). *p*-values < 0.05 were considered statistically significant.

## Results

The 8 CG centers and the 38 NCG centers submitted 498 and 1288 enrollment forms, respectively, during the study period. For the CG centers, 324 enrollments occurred prior to CG implementation and 174 enrollments took place post-CG implementation. Analysis of demographic data (Table 1) from both CG and NCG centers revealed no significant differences in patient characteristics.

**Table 1:** Patient demographics at NCG and CG centers

	Patients at NCG centers	Patients at CG centers	*p*-value
*N*, enrollments	1288	498	
Age, median (IQR)	13 (7.16)	13 (7.16)	0.331
Age groups, *N* (%)			0.833
Missing	9 (0.7)	3 (0.6)	
0–2	143 (11.1)	61 (12.2)	
3–5	116 (9.0)	47 (9.4)	
6–11	361 (28.0)	135 (27.1)	
12–17	512 (39.8)	205 (41.2)	
18+	147 (11.4)	47 (9.4)	
Race, *N* (%)			0.904
NH White	530 (41.1)	202 (40.6)	
NH Black	339 (26.3)	128 (25.7)	
Hispanic	345 (26.8)	135 (27.1)	
Other/missing	74 (5.7)	33 (6.6)	
Sex, *N* (%)			0.901
Missing	9 (0.7)	3 (0.6)	
Male	692 (53.7)	273 (54.8)	
Female	587 (45.6)	222 (44.6)	
Cause of kidney failure, *N* (%)			0.458
CAKUT	387 (30.0)	151 (30.3)	
GN	252 (19.6)	101 (20.3)	
PKD	36 (2.8)	19 (3.8)	
FSGS	212 (16.5)	78 (15.7)	
Ciliopathy	27 (2.1)	16 (3.2)	
Infarct	18 (1.4)	7 (1.4)	
Other	240 (18.6)	95 (19.1)	
Missing	116 (9.0)	31 (6.2)	

*NCG* non-ClearGuard, *NH* non-Hispanic, *CG* ClearGuard

**Fig. 1:**
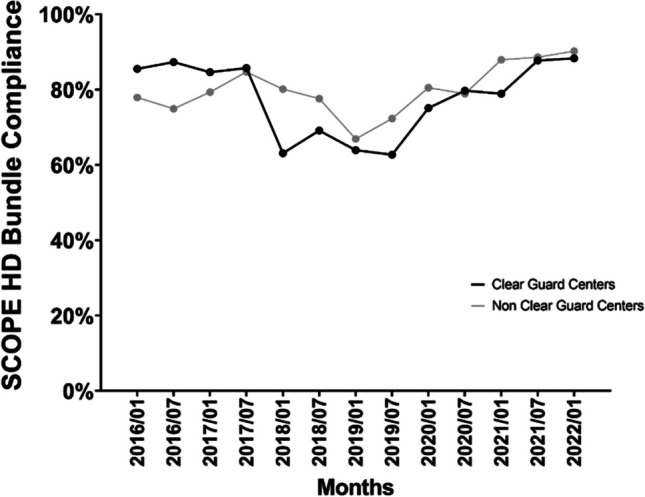
Adherence rate of ClearGuard and non-ClearGuard centers to SCOPE HD bundle over time

Both the CG and NCG centers showed increased compliance with the SCOPE HD care bundles over the 74-month period of observation (Fig. 1). There was a decrease in compliance (*p* < 0.001) from 2016 to mid-2018 for both CG and NCG centers; CG centers started with a higher level of compliance (85.5% vs. 77.9%, *p* < 0.001) in 2016 and saw steeper decline compared with NCG centers (*p* < 0.001). In mid-2018, compliance in both groups significantly increased (6-month OR [95% CI]: 1.31 (1.26, 1.35), *p* < 0.001), with similar rates of increase in both groups (*p* = 0.891).

**Fig. 2:**
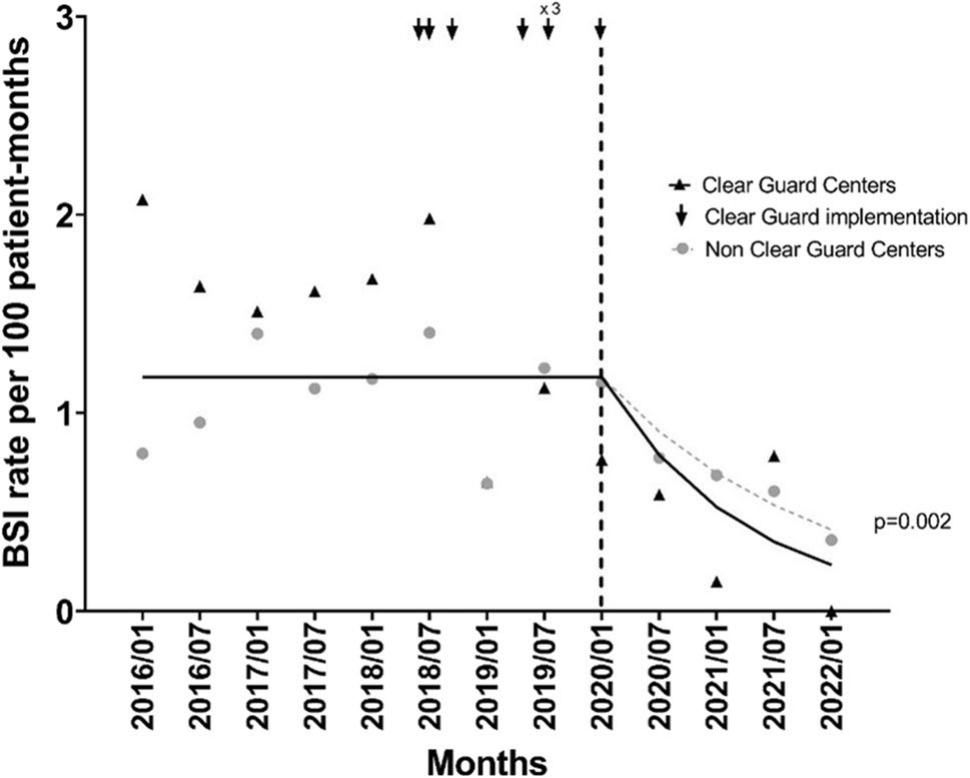
Change in HD catheter-associated bloodstream infection rates after January 2020

The CA-BSI rates decreased in both groups over time. The pre- and post-January 2020 rates for CG centers were 1.39 (1.08, 1.79) per 100 patient months and 0.46 (0.24, 0.88) per 100 patient months, while the pre- and post-implementation date CA-BSI rates for NCG centers were 1.10 (0.90, 1.35) per 100 patient months and 0.65 (0.46, 0.93) per 100 patient months. Based on the difference-in-difference analysis, there was no significant difference in CA-BSI rates between CG and NCG centers pre-January 2020 (*p* = 0.177); neither was there a significant change in CA-BSI rate over time during the pre-January 2020 period (*p* = 0.199). In an effort to provide the most conservative estimate of change in CA-BSI rate post-January 2020 for both CG and NCG centers, we modified the difference-in-difference model to fit a common intercept with no change over time in the pre-January 2020 time period. In turn, when comparing the decrease in the rates of HD CA-BSIs of the CG and NCG centers post-January 2020, the rate of decline experienced by the CG centers was significantly greater than the rate of decline for the NCG centers (*p* = 0.002) (Fig. 2). Table 2 provides the results of our difference-in-difference analysis post-CG if we chose an implementation date before January 2020. As noted in the table, our findings of a greater reduction in CA-BSI rates among CG centers were not dependent on our choice of implementation date.

**Table 2:** Post-implementation difference-in-difference results across candidate implementation dates

Implementation date	CG post-implementation rate ratio (95% CI)	NCG post-implementation rate ratio (95% CI)	*p*-value
7/1/2018	0.82 (0.72, 0.92)	0.88 (0.82, 0.92)	<0.001
1/1/2019	0.79 (0.67, 0.92)	0.86 (0.79, 0.94)	0.001
7/1/2019	0.73 (0.29, 0.90)	0.82 (0.73, 0.93)	0.001
1/1/2020	0.67 (0.49, 0.91)	0.77 (0.65, 0.91)	0.002

## Discussion

This study is the first to provide evidence that the use of ClearGuard antimicrobial caps is associated with a decrease in the rate of CA-BSI occurrence in children receiving long-term HD. There are a multitude of factors that may contribute to the development of CA-BSIs in HD patients and attention to many of these as elements within the SCOPE HD catheter care bundles have resulted in a significant decrease in the rate of infection of SCOPE participants. To that end, while the rate of infection decreased in both the CG and NCG centers during the study period, the decrease was greatest in those centers regularly using ClearGuard caps.

Studies in long-term adult HD patients have previously provided evidence of a significant correlation between the use of ClearGuard caps and a reduction of CA-BSIs in patients using a CVC for dialysis access. Two large, multicenter studies examined the impact of ClearGuard caps by studying > 4000 patients with CVCs, spanning over 500,000 catheter days [4, 5]. Those studies overwhelmingly demonstrated a decrease in CA-BSI rates for patients utilizing ClearGuard caps versus any other type of termination cap. While replicating the adult studies in children is challenging due to the lower volume of pediatric dialysis patients, conducting this analysis within the SCOPE collaborative did make data available from 46 centers and 1786 patients with CVCs for dialysis access.

Although our study volume was low compared to the adult experience, it is important to acknowledge that the ratio of CVCs to fistulae in pediatric centers is relatively high compared to that of adult centers, making the evaluation of strategies to decrease CA-BSIs in children exceedingly important. A variety of factors contribute to the frequent use of CVCs in children including the challenges associated with placement of a fistula in a young or small patient, as well as the intention to transplant quickly and not maintain dialysis for an extended period. Due to this intentional utilization of CVCs for this patient population, it is imperative to implement practices designed to minimize the risk of CA-BSI and the associated comorbidities in these children.

All pediatric patients evaluated in this study were enrolled in the SCOPE collaborative. Achievement of a progressive decrease in the rate of CA-BSIs in HD patients via implementation of standardized infection prevention practices and care bundles remains a primary goal of the collaborative. As previously published, the SCOPE collaborative saw a decrease in CA-BSI infection rates from 3.3 to 0.8 per 100 patient months associated with increased bundle adherence between June 2013 and May 2017, among centers that provided baseline infection data between June 2012 and May 2013 [6].

The success associated with the implementation of the care bundles is in large part due to compliance with the bundle elements. Review of SCOPE data has provided evidence that over time, compliance with the SCOPE HD care bundles increased among all SCOPE centers, both those designated as CG centers and NCG centers for this study (Fig. 1). While compliance increased similarly for both groups with an associated decrease in CA-BSI rates experienced by both, the CG group showed a more significant decrease in CA-BSI rates post-CG implementation (Fig. 2). This finding suggests that CG implementation has a synergistic effect with the bundle elements on CA-BSI prevention and may, in addition, potentially protect against any bundle non-adherence.

In CG centers surveyed for this study, the initial supply cost, determined by contracting, was perceived to be higher than the cost associated with other types of termination caps for a variety of reasons, including the base cost of the caps and the fact that CG caps are not reusable (each entry into the HD line requires a new cap to be placed). However, SCOPE centers implementing the CG caps report a potential decrease in overall spending by further reducing CA-BSIs and associated hospitalizations, as well as eliminating the need for other costly antimicrobial interventions such as antimicrobial HD catheter locks. Further study of this issue will provide important cost benefit information.

As for practice changes and administrative support, it is clear that the HD population makes up a small subset of patients in a pediatric institution. The implementation of CG caps mandates that HD CVCs are cared for differently than all other central lines within a hospital system. Thus, the case for change must be very strong to outweigh the risks associated with implementing a high-risk, low-volume practice change.

There are limitations to this study, the most important being the limited number of SCOPE centers and patients who have used CG caps for more than 2 years. In addition, the data are observational. It is also possible that other unmeasured confounding factors associated with both CG centers and the reduction in CA-BSI rate explain these results. However, the availability of comparison centers that have not used CG, in addition to the experience of the SCOPE centers in collecting and submitting infection-related data and data pertaining to infection prevention activities, is a definite strength which has contributed to the success of the study.

In conclusion, this study of pediatric implementation of ClearGuard antimicrobial caps for CA-BSI prevention in children on long-term HD provides compelling evidence of an effective strategy to complement established infection prevention practices. Additional data is needed regarding the efficacy of CG caps to solidify the justification for implementation of cost and practice changes on a more widespread basis.

### Supplementary information

Below is the link to the electronic supplementary material.

Graphical abstract (PPTX 162 KB)

## Data Availability

The datasets generated during and/or analyzed during the current study are subject to SCOPE participation agreements and are not publicly available.
