# Correction: Use of ClearGuard HD caps in pediatric hemodialysis patients

**DOI:** 10.1007/s00467-024-06393-7

**Published:** 2024-05-08

**Authors:** Amy Nau, Troy Richardson, Diana Cardwell, Jennifer Ehrlich, Jyothsna Gattineni, Melisha Hanna, Mahima Keswani, Emily Neibauer, Kelly Nitz, Raymond Quigley, Michelle Rheault, Rebekah Sims, Mayna Woo, Bradley A. Warady

**Affiliations:** 1 Division of Pediatric Nephrology, Children’s Mercy Kansas City, Kansas City, MO USA; 2 Children’s Hospital Association, Lenexa, KS USA; 3 Division of Pediatric Nephrology, Children’s Health Dallas, Dallas, TX USA; 4 Division of Pediatric Nephrology, Stead Family Children’s Hospital, University of Iowa, Iowa City, IA USA; 5 Division of Pediatric Nephrology, Children’s Hospital Colorado, Aurora, CO USA; 6 Division of Pediatric Nephrology, Ann and Robert H. Lurie Children’s Hospital, Chicago, IL USA; 7 Division of Pediatric Nephrology, Children’s Hospital of Wisconsin, Milwaukee, WI USA; 8 Division of Pediatric Nephrology, Children’s Minnesota, Minneapolis, MN USA; 9 Division of Pediatric Nephrology, Children’s of Alabama, Birmingham, AL USA; 10 Division of Pediatric Nephrology, Stanford Medicine-Children’s Health, Lucile Packard Children’s Hospital, Palo Alto, CA USA

**Correction: Pediatric Nephrology**

10.1007/s00467-023-06273-6

The article “Use of ClearGuard HD caps in pediatric hemodialysis patients”, written by Amy Nau, Troy Richardson, Diana Cardwell, Jennifer Ehrlich, Jyothsna Gattineni, Melisha Hanna, Mahima Keswani, Emily Neibauer, Kelly Nitz, Raymond Quigley, Michelle Rheault, Rebekah Sims, Mayna Woo and Bradley A. Warady, was originally published electronically on the publisher’s internet portal on 25.01.2024 without open access. With the author(s)’ decision to opt for Open Choice the copyright of the article changed on 22.04.2024 to © The Authors 2024 and the article is forthwith distributed under a Creative Commons Attribution 4.0 International License, which permits use, sharing, adaptation, distribution and reproduction in any medium or format, as long as you give appropriate credit to the original author(s) and the source, provide a link to the Creative Commons licence, and indicate if changes were made. The images or other third party material in this article are included in the article's Creative Commons licence, unless indicated otherwise in a credit line to the material. If material is not included in the article's Creative Commons licence and your intended use is not permitted by statutory regulation or exceeds the permitted use, you will need to obtain permission directly from the copyright holder. To view a copy of this licence, visit http://creativecommons.org/licenses/by/4.0/.

