# Supplementary material for: Use of ClearGuard HD caps in pediatric hemodialysis patients
Source: Pediatr Nephrol. 2024 Jan 25;39(7):2171–5. doi: 10.1007/s00467-023-06273-6 (PMC11147870; doi:10.1007/s00467-023-06273-6)
Supplement: Supplementary file 1 — Graphical abstract (PPTX 162 KB) [file 467_2023_6273_MOESM1_ESM.pptx]

## Slide 1
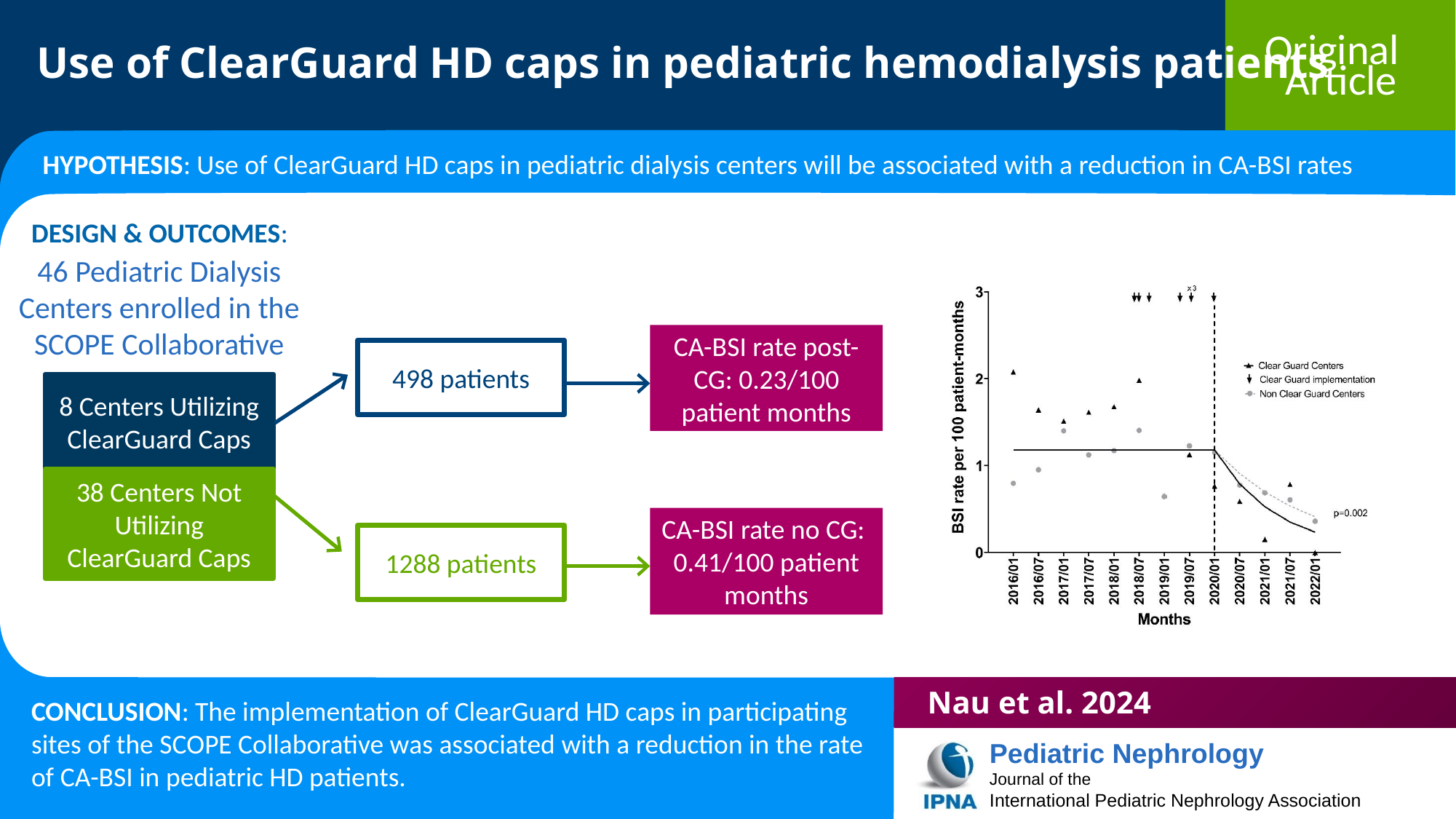

Use of ClearGuard HD caps in pediatric hemodialysis patients
HYPOTHESIS: Use of ClearGuard HD caps in pediatric dialysis centers will be associated with a reduction in CA-BSI rates
DESIGN & OUTCOMES:
46 Pediatric Dialysis Centers enrolled in the SCOPE Collaborative
CA-BSI rate post- CG: 0.23/100 patient months
498 patients
8 Centers Utilizing ClearGuard Caps
38 Centers Not Utilizing ClearGuard Caps
CA-BSI rate no CG: 0.41/100 patient months
1288 patients
Nau et al. 2024
CONCLUSION: The implementation of ClearGuard HD caps in participating sites of the SCOPE Collaborative was associated with a reduction in the rate of CA-BSI in pediatric HD patients.
